# Intergenerational Effect of Early Life Exposure to Permethrin: Changes in Global DNA Methylation and in *Nurr1* Gene Expression

**DOI:** 10.3390/toxics3040451

**Published:** 2015-11-19

**Authors:** Laura Bordoni, Cinzia Nasuti, Maria Mirto, Fabio Caradonna, Rosita Gabbianelli

**Affiliations:** 1 School of Advanced Studies, University of Camerino, Via Gentile III da Varano, Camerino 62032, Macerata, Italy; E-Mail: laura.bordoni@unicam.it; 2 Gabbianelli Rosita, School of Pharmacy, University of Camerino, Via Gentile III da Varano, Camerino 62032, Macerata, Italy; E-Mail: cinzia.nasuti@unicam.it; 3 Department of Biological Chemical and Pharmaceutical Sciences and Technologies (STEBICEF, Unit of Cellular Biology), University of Palermo, Palermo 90128, Italy; E-Mails: mariamirto87@gmail.com (M.M.); fabio.caradonna@unipa.it (F.C.)

**Keywords:** permethrin, intergenerational effect, *Nurr1*, global DNA methylation

## Abstract

Environmental exposure to pesticides during the early stages of development represents an important risk factor for the onset of neurodegenerative diseases in adult age. Neonatal exposure to Permethrin (PERM), a member of the family of synthetic pyrethroids, can induce a Parkinson-like disease and cause some alterations in striatum of rats, involving both genetic and epigenetic pathways. Through gene expression analysis and global DNA methylation assessment in both PERM-treated parents and their untreated offspring, we investigated on the prospective intergenerational effect of this pesticide. Thirty-three percent of progeny presents the same *Nurr1* alteration as rats exposed to permethrin in early life. A decrease in global genome-wide DNA methylation was measured in mothers exposed in early life to permethrin as well as in their offspring, whereas untreated rats have a hypermethylated genomic DNA. Further studies are however needed to elucidate the molecular mechanisms, but, despite this, an intergenerational PERM-induced damage on progenies has been identified for the first time.

## 1. Introduction

Early life environmental exposure to pesticides represents an important risk factor for the development of neurodegenerative diseases in adult age [1,2,3]. Understanding which, when and how neurotoxicants cause long-term effects on health is crucial, because it would increase our knowledge on the mechanisms associated with the aetiology of neurodegenerative processes and might permit one to identify healthy procedures devoted to avoiding or mitigating exposure during developmental stages.

Pyrethroid pesticides exposure, as well as exposure to other neurotoxicants, are associated with neurodegenerative diseases because they can interfere with mitochondrial function, increase oxidative stress and alpha-synuclein aggregation by genetic and epigenetic mechanisms [4,5,6,7]. Permethrin (PERM) is a member of the family of synthetic pyrethroids, which is not only used in agriculture but is also largely employed as an insecticide for indoor residential treatment (*i.e.*, carpets, kitchen worktops and other treated wood furniture), mosquitos control, occupational take-home exposures, pets, personal care products [8]. PERM was the first synthetic photostable pyrethroid to be used in agriculture and the most used pyrethroid in USA. It has a half-life of less than 28 days in soil and about 10 days on plants and its use is forbidden in seas, near rivers and lakes because of its high toxicity to fish (Agency for Toxic Substance and Disease Registry, 2005). The wide exposure to humans is demonstrated by the presence of a PERM metabolite, 3-phenoxybenzoic acid (3-PBA), in urine in 98% of the population; moreover, the higher level of 3-PBA in children compared to adults represents a worrisome aspect for health of future generations.

In our animal model, we previously demonstrated that early life exposure (from postnatal day 6 to 21) to a low dose of PERM induces a Parkinson-like disease. In particular, in the striatum of adult and old rats, changes in *Nurr1* gene expression and reduced dopamine (DA) level together with its accelerated turnover were observed [9,10,11,12]. Moreover, oxidative stress, high plasma NO production, protein and lipid oxidation, low GSH levels were measured [13]. In the same model, we demonstrated that PERM accumulates in the brain later after the end of treatment, and that early life exposure can modify DNA methyltransferases and alfa-synuclein, suggesting that PERM might mediate genetic and epigenetic modifications leading to development of neurological disorders with some typical features of Parkinson’s-like disease [7,9,10,12,14,15].

Since epigenetic mechanisms can mediate the interaction between environmental and genetic factors and provide a “cellular memory” that maintains a disease status over one’s lifetime and to the next generations [16]; here, we present data related to the intergenerational effect of early life PERM exposure. In particular, we analysed if the offspring (F1 generation) of rats exposed to a low dose of PERM from postnatal day 6 to 21, present alterations in *Nurr1* gene expression as previously observed in male rats. Moreover, global DNA methylation was analyzed in untreated, early life exposed mothers and offspring (F1 generation).

## 2. Experimental Section

### 2.1. Materials

All reagents were of analytical grade and were obtained from Sigma Chemical Co. (Balcatta, WA, USA). Technical grade (75:25, trans:cis; 94% purity) 3-phenoxybenzyl-(1*R*,*S*)-cis,trans-3-(2,2-dichlorovinyl)-2,2-dimethylcyclopropanecarboxylate, Permethrin was a generous gift by Dr. A. Stefanini of ACTIVA, Milan, Italy.

### 2.2. Animals

Male and female Wistar rats aged about 90 days weighing 250–270 g were obtained from Charles River (Calco, Lecco, Italy). The rats were housed in plastic cages (five rats/cage) in a temperature controlled room (21 ± 5 °C) and fed with a laboratory diet and water ad libitum. The light/dark cycle was from 7 a.m. to 7 p.m. Animal used in this study complied with European Directive (2010/63/EU) related to the protection of animals used for scientific studies. Rat pups born in our laboratory from primiparous dams were used in the study. The parturition day was set as Post Natal Day 0 (PND0). On PND1, all litters were checked for the presence of gross abnormalities, sexed and weighed. Two male and one female pup were assigned to each dam until weaning (PND21). No cross-fostering was employed. At two days of age, litters were casually assigned to two experimental groups named control and treated ones.

### 2.3. Treatment and Experimental Design

PERM was solubilized in corn oil and the animals were gavaged with intragastric tube (4 mL/kg) at a dose of 1/50 of LD50 corresponding to 34.05 mg/kg (Agency for Toxic Substance and Disease Registry, 2005). The dosage was chosen considering that NOAEL (no observed adverse effect level) for PERM is 25 mg/kg. The compound was administered daily in the morning from PND6 to PND21. Control group was administered with vehicle (corn oil, 4 mL/kg) on a similar schedule. The volume of solutions to be administered were adjusted daily based on body weight. On PND21, the pups were weaned, housed two per cage and assigned to two different experiments.

Experiment 1: at the adolescent age (PND < 90 days), six male rats treated with PERM and six male rats not treated were sacrificed by exposure to CO_2_. The striatum from each rat was isolated from the brain and immediately placed in liquid nitrogen and stored at −80 °C.

Experiment 2: at the age of four months, females treated with PERM were mated with males treated with PERM and females not treated with PERM were mated with males not treated (no siblings). Their F1 offspring were the focus of the present study. The final F1 sample sizes were nine pups from the treated F0 group and two pups from the control F0 group, and F1 generation was not submitted to any treatment. At the adolescent age (PND 65), five males and four females from treated F0 group and three males and three females from the control F0 group were sacrificed by exposure to CO_2_. The striatum from each rat was isolated from the brain and immediately placed in liquid nitrogen and stored at −80 °C.

### 2.4. Nucleic Acids Extraction

Genomic DNA was extracted from striatum obtained from each rat, by DNazol Reagent (Life Technologies, Thermo Fisher Scientific Inc., Waltham, MA, USA) according to the manufacturer’s instructions. Total RNA was also extracted from striatum of each sample by using a RNA Isolation kit (NucleoSpin RNA Purification Kit, Macherey-Nagel, GmbH & Co. KG, Düren, Germany). DNA and RNA purity and quantity were checked by spectrophotometric analysis (OD260/280; OD260/230) with K2800 Nucleic Acid Analyzer (Beijing Kaiao Technology Development Co., Ltd., Beijing, China).

### 2.5. MeSAP-PCR

Methylation Sensitive Arbitrarily Primed Polymerase Chain Reaction (MeSAP-PCR) is a technique based on a methylation-sensitive enzymatic digestion, followed by two consecutive PCR reactions. It is able to identify different methylation patterns in GC-rich genomic DNA regions [17]. Genomic DNA from each subject was digested for 16 h at 37 °C with RsaI (10 U), in a final volume of 50 μL, obtaining the mono-digested DNA (M). Then, half of this product was processed with a second digestion with HpaII (5 U) overnight, gaining the double-digested DNA (D). Contrary to the first digestion, the second one is methylation-sensitive: HpaII cuts its restriction site only if the cytosines are un-methylated; then, enzymes heat-inactivation (65 °C for 30 min) was performed. An Arbitrarily Primed PCR (AP-PCR) was performed with both M and D from each sample. This AP-PCR consists of two consequent PCR reactions: AP1 and AP2. The same arbitrary oligonucleotide (5′-AAC TGA AGC AGT GGC CTC GCG-3′) was used as primer for both amplifications: it is GC-rich (more than 50%) and has a CGCG-3′ tail, so it can preferentially anneal and amplify GC-rich DNA sequences. Different temperature programs and MgCl_2_ concentrations were used to keep the AP1 non-specific and AP2 specific. The reaction mix for AP1 was: MgCl_2_ 4.5 mM; dNTP 0.2 mM; arbitrary primer 10 μM, Taq DNA Polymerase (Roche) 0.8 U in a final volume of 25 μL. AP1 thermal profile was: initial denaturation of 94 °C for 5 min; 4 cycle of 94 °C for 30 s, 40 °C for 60 s, 72 °C for 90 s. AP2 reaction mix was completed by adding MgCl_2_ 4.5 mM, dNTP 0.2 mM and Taq polymerase 2.5 U, in a final volume of 75 μL. AP2 thermal profile was: 29 cycles of 94 °C for 60 s, 60 °C for 60 s, 72 °C for 120 s.

The amplification products were analyzed with a high resolution polyacrylamide gel (12%), running for 5 h at 200 V in TBE 1× buffer. After gel treatment with GelRed (Biotium Inc., Hayward, CA, USA) for 40 min, a DNA fingerprinting was revealed on Chemidoc (Bio-rad Inc., Hercules, CA, USA). With a densitometric analysis and the usage of specific software (SigmaGel, Jandel Scientific, Erkrath, Germany), it is possible to obtain a graph that clearly shows the differences in genomic methylation between each sample. Comparing for each sample, the M fingerprinting with the D one, it is possible to show and semi-quantify different methylation patterns.

### 2.6. Gene Expression Analysis

The expression of target gene was assessed by RT-PCR. RNA was transcribed *in vitro* to cDNA using PrimeScriptTM RT reagent Kit (Takara Bio, Inc., Kusatsu, Japan) according to the manufacturer’s instructions. The following specific sense and antisense primers were designed considering gene and mRNA sequences available online [18] and then purchased from Sigma Chemical Co. (USA): β-Actin, TAAAGACCTCTATGCCAACACAGTGC (forward) and AGAGTACTTGCGCTCAGGAGGAG (reverse); *Nurr*-1, GGTTTCTTTAAGCGCACGGTG (forward) and TTCTTTAACCATCCCAACAGCCAG (reverse). The primers used here were designed as previously reported in [9]. β-actin was considered as a relatively invariant internal reference, and its amplification was performed in parallel. RT-PCR analysis was executed in a total volume of 20 μL containing 50 ng of template cDNA, 0.5 μM sense and antisense primers and 10 μL of iQ SYBR Green Supermix (Bio-Rad Inc.), by using a CFX Connect Real-Time PCR Detection System (Bio-Rad Inc.). The real-time PCR program was: initial denaturation at 95 °C for 3 min; and 40 cycles at 95 °C for 30 s, 63 °C for 30 s and 72 °C for 1 min, followed by a melting curve (65 to 95 °C, increment 0.5 °C, for 5 s). Relative mRNA expression on each tissue sample was quantified according to the ΔΔ*C_t_* method. The experiment was run three times in triplicate.

### 2.7. Statistical Analysis

Data are presented as media ± SD. The data were analyzed by means of a Student *t*-test. Differences were considered significant at *p* value ≤ 0.05.

## 3. Results and Discussion

### 3.1. General Findings

No significant changes in body weight of early life PERM-treated rats compared to controls were measured throughout their life in mothers and in offspring (data not shown).

### 3.2. Nurr 1 Gene Expression in Young Rats

**Figure 1:**
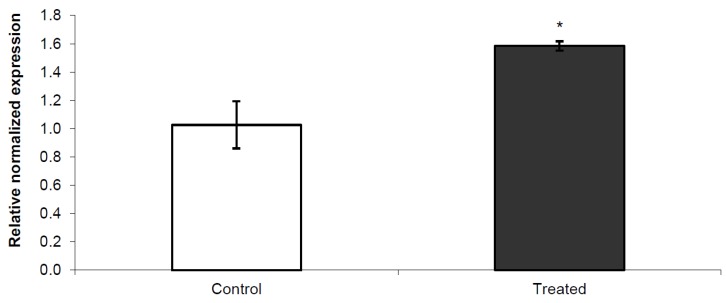
qPCR to quantify relative changes in *Nurr1* expression in striatum of young rats exposed to permethrin treatment in early life. All expression values were normalized to the value of β-actin gene used as an internal control. * *p* < 0.05 *vs.* control.

Figure 1 shows *Nurr1* gene expression in adolescent rats exposed to PERM pesticide during early life from PND6 to PND21. A significant increase in *Nurr1* gene expression was measured in treated animals compared to control ones.

### 3.3. Nurr1 Gene Expression in F1 Offspring

**Figure 2:**
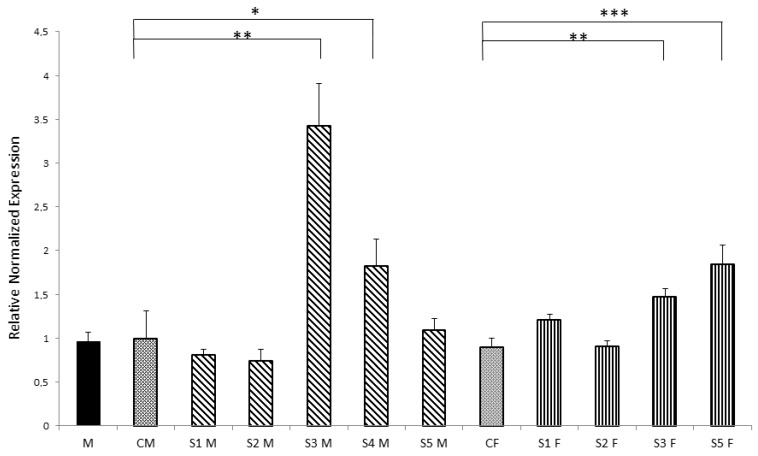
qPCR to quantify *Nurr1* expression relative changes in striatum of control, early life treated mother and offspring (F1 generation, untreated rats). All expression values were normalized to the value of β-actin gene used as an internal control. * *p* < 0.05; ** *p*< 0.01; *** *p* < 0.001 *vs.* matched sex control group.

Figure 2 shows *Nurr1* gene expression measured in offspring born from parents treated in early life. Both mothers and fathers were treated in early life, while offspring were untreated. Comparing the average of each group by sex, no significant differences between F1 control group and F1 offspring group from treated parents were observed (data not shown). Instead, comparing single rats with their matched sex control group, 40% males and 50% females, an increase in *Nurr1* gene expression was observed. This increase was higher in males (S3M: 3.43, *p* = 0.0028; S4M: 1.83, *p* = 0.032) than females (S3F: 1.64, *p* = 0.0027; S5F: 2.05, *p* = 0.00023) as displayed in Figure 2.

### 3.4. Global DNA Methylation Assessed by MeSAP-PCR

With reference to MeSAP-PCR, banding patterns obtained after amplification of DDD and MDD will be the same only when CpG-rich sequences are hypermethylated; on the contrary, banding patterns will result differently in direct relationship with hypomethylation. The differences can be: (i) presence or absence of additional bands (appearance/disappearance) representing other fragments with different molecular weight or (ii) variations in the intensity of pre-existing bands (attenuation/intensification), depending on the co-migration of missing or additional fragments with the same molecular weight but with different sequence.

Table 1 shows genomic methylation changes observed in DNA of untreated rats, mothers (treated in early life) and offspring F1 generation (untreated).

**Table 1:** Genomic methylation changes observed in DNA of untreated rats, mother (early life treated) and offspring F1 generation (untreated). NTM (untreated male controls) and NTF (untreated female controls) SM (male son rat) SF (female daughter rat).

Sample	Total of Variations	Mean Value ± SD
**NTM (Untreated)**	3	3.5 ± 0.707
**NTF (Untreated)**	4	
**M1 (Mother)**	8	7 ± 1.732
**M2 (Mother)**	8	
**M3 (Mother)**	5	
**S1M**	4	7 ± 2.00
**S2M**	8	
**S3M**	6	
**S4M**	9	
**S5M**	8	
**S1F**	5	6.5 ± 2.38
**S2F**	10	
**S3F**	6	
**S5F**	5	

**Figure 3:**
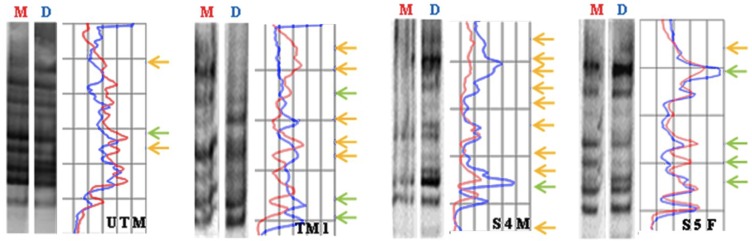
Methylation sensitive Arbitrarily Primed PCR of four representative samples. UTM = Untreated Male; TM1 = Early life treated mother; S4M = Male F1 Offspring; S5F = Female F1 Offspring. M= Mono-digested DNA; D= Double-digested DNA. Arrows in **yellow** indicate the disappearing/appearing of bands; arrows in **green** indicate attenuation/intensification of bands.

Global methylation decreased similarly in early life exposed mothers and in untreated offspring (F1 generation). MeSAP-PCR analysis of representative samples shows that DNA of mothers and untreated offspring is hypomethylated compared to the control (untreated) (Figure 3).

### 3.5. Discussion

*Nurr1*, an orphan nuclear receptor belonging to NR4A subfamily, is a transcription factor expressed in the embryonic ventral midbrain. It is critical for the development and maintenance of DA neurons because it activates the transcription of tyrosine hydroxylase (TH) and DA active transporter (DAT) genes, whose corresponding proteins are required for biosynthesis and storage of DA, respectively. It is also known that D2-like receptors interact with *Nurr1* via extracellular signal-regulated kinase (ERK) signaling, that, in turn, is critical for the activation of *Nurr1* and for dopaminergic (DArgic) neuronal development [19]. For its function on dopaminergic neurons, *Nurr1* has been studied in the midbrain autopsy of Parkinson’s disease (PD) patients, where an unbalanced expression was observed [20]. Moreover, an experimental rat model of PD, obtained by treating rats during early life with PERM, showed a change in *Nurr1* regulation with aging [9,10]. In the present study, we observed that neonatal exposure to PERM during brain development, leads to an increase in *Nurr1* gene expression in adolescent age (Figure 1) and that 44.4% of untreated offspring, generated by early life exposed rats, have a similar variation in *Nurr1* gene expression (Figure 2). Considering that PERM is able to cross the blood brain barrier, it can accumulate inside the brain after the end of treatment, and that *in silico* studies have identified several sites of binding between *Nurr1* and PERM [7,20], and a direct interaction between PERM and *Nurr1* might be suggested in treated rats. Furthermore, since dopamine level decreases 10 times from adolescent to old age together with an imbalance in the redox system [11,12,21], other disturbing factors, which indirectly perturb the neuronal homeostasis, should also be considered. To explain the intergenerational effect on *Nurr1* in F1 generation, epigenetic modifications should be considered. Epigenetic changes, like DNA methylations and histone modifications, are tissue specific modifications able to perturb gene expression without modifying the gene sequence and can be transferred to the progenies. Increase in DNA methyltransferases activity (DNMTs), the enzymes responsible for DNA methylation, was measured in this animal model [7], in agreement with the literature where several evidences on epigenetic modulation induced by pesticides are described [22,23,24,25].

Our data also underline an intergenerational alteration in *Nurr1* expression in F1 generation from PERM-treated parents. In particular, the effect is stronger in male rats than in females. 

Moreover, if we analyze data on global DNA methylation, we observe that hypomethylation measured in the mothers exposed to PERM during early life, is detectable also in the offspring, and that, on the contrary, DNA of untreated rats is hypermethylated.

This result, apparently not consistent with higher DNMT expression levels detected in adolescent PERM-treated rats (unpublished data), could be explained by an indirect unbalanced methylation effect promoted by PERM-induced reactive oxygen species (ROS) [26]. In fact, it has been described that ROS are able to induce genomic hypomethylation of DNA by the formation of 8-hydroxy-2′-deoxyguanosine (8-OHdG), which can lead to DNA hypomethylation. On the other hand, ROS may induce site-specific hypermethylation via the up-regulation of expression of DNA methyltransferases (DNMTs) [27]. We believe that PERM-induced ROS are the real actors of genomic hypomethylation and of the contemporary DNMT up-regulation. This hypothesis is in agreement with previous studies where an increase in oxidative stress in striatum of PERM-treated rats was observed [5,10,11,28,29]. In particular, these rats showed lipid and protein oxidation, DNA damage and lower levels of GSH in the striatum [5,10,11,28,29].

In support of the above is that global DNA hypomethylation was also reported in the population with high blood levels of pesticides [24,30,31]. The most interesting finding of the present study is the intergenerational effect of PERM. In particular, the same alteration in DNA methylation was seen in treated parents and in unexposed offspring. This alarming intergenerational effect of PERM must be better investigated in further studies.

The clinical effects linked with the observed alteration are not known yet. Nevertheless, we can expect that the untreated F1 generation could develop the same clinical impairment observed in their PERM-treated parents [11,12,13,29]. Knowledge on intergenerational heritage could open a path for recognizing new risk factors associated with the development of dopaminergic neurodegeneration.

## 4. Conclusions

In conclusion, we here demonstrate for the first time that early life exposure to PERM can be associated with intergenerational effects on offspring. Further studies on PERM-induced ROS and on *Nurr1* promoter methylation and histone modifications will be required to define the mechanisms associated with the change in *Nurr1* gene expression.
